# Foundations for a Personalized Psycho-Oncology: The State of the Art

**DOI:** 10.3390/jpm14090892

**Published:** 2024-08-23

**Authors:** Giampaolo Perna, Eleonora Pinto, Alessandro Spiti, Tatiana Torti, Michele Cucchi, Daniela Caldirola

**Affiliations:** 1 Department of Biological Sciences, Humanitas University, 20089 Milan, Italy; daniela.caldirola@hunimed.eu; 2 IRCCS Humanitas Research Hospital, 20089 Milan, Italy; alessandro.spiti@mc.humanitas.it (A.S.); michele.cucchi@humanitas.it (M.C.); 3 Veneto Institute of Oncology IOV–IRCCS, 35128 Padua, Italy; eleonora.pinto@iov.veneto.it; 4 ASIPSE School of Cognitive-Behavioral-Therapy, 20124 Milan, Italy; tatiana.torti@asipse.it

**Keywords:** personalized medicine, psycho-oncology, psychiatric disorders, psychological disorders, psychotherapeutic interventions, drug–drug interactions, inflammatory biomarkers, cancer-related psychological distress, personalized follow-up, tailored mental health care

## Abstract

Personalized psycho-oncology represents a major challenge for the holistic care of cancer patients. It focuses on individualized psychotherapeutic and psychiatric interventions to address specific psychological needs. This narrative review summarizes the current literature on personalized psycho-oncology and highlights the prevalence and impact of psychiatric/psychological disorders in cancer patients. Personalized approaches, including tailored interventions and interdisciplinary collaboration, have been shown to be effective in improving mental health and overall quality of life. The integration of inflammatory biomarkers into treatment plans is a promising but challenging way to alleviate mental health problems. In addition, there is a need for specific diagnostic tools and treatment guidelines that take into account the specific psychological impact of different types of cancer. Future research should aim to refine these personalized strategies, improve diagnostic accuracy, and evaluate the cost-effectiveness of these interventions to improve both the psychological well-being and treatment outcomes of cancer patients.

## 1. Introduction

### 1.1. Personalized Approach: Definition in Medicine and Its Application in Oncology

Personalized medicine (PM) is a medical practice that tailors the treatment and prevention of disease to individual genetic, molecular, clinical, physiological, and environmental characteristics. This approach represents a paradigm shift away from the one-size-fits-all model towards providing the right treatment to the right person at the right time, improving therapeutic outcomes and minimizing side effects [1]. The focus is on using molecular diagnostics to integrate clinical diagnosis and therapy to improve the stratification of treatment pathways based on disease susceptibility, etiology, or prognosis [2].

The patient-centered approach complements personalized medicine by involving patients in their treatment and respecting their beliefs, values, and preferences. It promotes a holistic view of the individual as a biopsychosocial being and supports a therapeutic alliance between patient and physician that enables patient participation in the decision-making process [3,4]. Despite its widespread use, there are still challenges in effectively operationalizing this approach, particularly in terms of communication [5].

PM also represents an innovative approach for the treatment and prevention of cancer in oncology. This method takes into account the unique genetic characteristics, tumor microenvironment, lifestyle factors, and existing health status of each cancer patient. By focusing on the specific genetic mutations that drive tumor growth and the tumor’s immunological environment, PM aims to develop tailored therapies that effectively fight cancer while minimizing side effects to improve patients’ quality of life [6]. Ultimately, this approach aims to provide superior, individualized patient care that extends its benefits to the treatment of psychological and psychiatric issues in cancer patients.

### 1.2. Psycho-Oncology as Part of Personalized Oncology and Its Definition

Psychiatric disorders and psychological distress are particularly common in cancer patients, which has a significant impact on their overall prognosis and survival. Studies show that almost half of cancer patients suffer from some form of psychiatric/psychological disorder, with adjustment disorders being the most common, affecting up to 32% of patients [7,8]. In addition, about 14–25% of cancer patients experience major depressive disorder, which significantly affects their quality of life and treatment outcomes [9].

The presence of psychiatric/psychological comorbidities in cancer patients is associated with an increased mortality rate. Psychiatric/psychological patients have a higher mortality rate from cancer compared to the general population, despite having a similar incidence rate of cancer [10]. This disparity is partly due to delays in cancer diagnosis and limited access to specialized cancer treatments. These patients are more likely to have an advanced stage of cancer and less likely to receive surgical, radiotherapy, and chemotherapy treatments [10].

The chronic distress associated with a cancer diagnosis leads to physiological changes, such as increased cortisol levels and immune changes, which can exacerbate psychiatric/psychological symptoms and contribute to a poorer prognosis [11]. Although the current state of knowledge on the direct link between mental state and survival rates in cancer patients needs to be further explored, the impact of mental imbalance, such as reduced adherence to treatment, has a significant impact on the likelihood of outcomes, from screening to subsequent steps in clinical care [12]. These findings highlight the importance of early detection and treatment of mental health problems in cancer patients to improve overall outcomes and increase survival. Addressing these needs of cancer patients is essential not only for improving their mental health but also for improving oncologic outcomes.

A recent analysis of the science supporting personalized oncology has shown that oncology is able to pursue the strategy of personalization even when the tools of genomic sequencing are not yet effective [13]. Indeed, oncology promotes appropriate disease management based on individual variations in a patient’s profile, both in terms of tumor (genetic, molecular) and patient characteristics (e.g., environment, lifestyle).

As sciences that deal with the individual characteristics and variability of patients, psychology and psychiatry support oncology by exploring individual habits, cognitions, beliefs, behaviors, and potential mental health symptoms or disorders. As a result, patients are increasingly involved in treatment assessments and decision-making processes, with psychological variables being investigated as potential factors influencing cancer risk, progression, prevention, and detection. By examining the links between psychological and oncologic outcomes, focusing on compliance, coping, and resilience, psychology began to support oncologic intervention.

The study of these connections between physical, psychological, behavioral, social, and ethical aspects of cancer is known as psycho-oncology. It is a specialized discipline that integrates psychiatric and psychological knowledge. Nevertheless, psycho-oncology should be a structured application of this scientific methodology: like other disciplines concerned with the healthcare system and patient care, psycho-oncology should have a grounded approach. The use of combined psychological and psychiatric knowledge in psycho-oncology can be enhanced by understanding the biological consequences of the tumor environment to make specific predictions about the health of cancer patients [2].

By conceptualizing health as a process that includes different ways of predicting pathologies and/or effects of actions at the organic level as well as at the level of interaction between the roles involved in the management of the cancer diagnosis (healthcare professionals, patients, caregivers, community) [14], the integration of knowledge can be directed towards the patient’s health. One of the first steps in achieving patient health without standardization is to structure an identifiable and measurable psycho-oncological intervention.

### 1.3. Introduction to the Key Question for a Founded Psycho-Oncology

The field of psycho-oncology has made significant progress over the years. Within the World Psychiatric Association (WPA), there has been a dedicated section since the late 1980s that focuses on the integration of psychiatry and behavioral sciences into oncology and palliative care [15].

This section has played a critical role in advancing research, clinical care, and training in psycho-oncology, promoting the recognition of psychosocial cancer care as a universal human right, and emphasizing the need to integrate a personalized approach into routine cancer care. Therefore, this narrative review aimed to identify the proper basis for a founded, personalized psycho-oncology. Specifically, our goals were to offer a comprehensive overview of recent advancements in this field, identify gaps in the current literature that require further research, and highlight the significance of personalized psychotherapeutic and psychiatric interventions tailored to the unique needs of cancer patients. Additionally, we emphasized the critical role of integrated approaches and interdisciplinary collaboration in optimizing mental health outcomes and overall quality of life for patients.

## 2. Materials and Methods

Against this background, the basis for personalized psycho-oncology can be identified in the following main areas: psychiatry, psychotherapy, psychopharmacology, and the role of healthcare professionals involved in the treatment process.

To achieve the aim of this paper, a comprehensive search was conducted in the main bibliographic databases (PubMed, Cochrane Library, EMBASE) using different combinations of these keywords and medical subject heading terms: “oncology”, “neoplasm”, “patient-centered approach”, “personalized medicine”, “precision medicine”, “individualized approach”, “psychiatry”, “psychotherapy”, “psychopharmacology”, and “health- professionals”.

The inclusion criteria were the following: peer-reviewed articles, including systematic reviews, meta-analyses, clinical trials, and research studies that provide empirical data or comprehensive reviews related to personalized psycho-oncology and are written in English to ensure accessibility and comprehensibility. Book chapters were also considered if they were written in English and contained the above information. The search was limited to articles published in the last eight years (2016–2024) to ensure that the latest research was included. The patient population criteria included studies involving adult cancer patients.

The exclusion criteria were as follows: Studies that were case reports, editorials, letters to the editor, and non-peer-reviewed articles; articles published in languages other than English or in gray literature; studies published outside the specified time period, unless they were basic or highly cited papers; and studies that involved only pediatric patients under the age of 18 to maintain the focus on adult cancer patients.

In addition, from the articles found in the initial search, further references were identified through a manual search of the cited sources.

All authors discussed the search results step by step and reached a consensus on the relevance and inclusion of the identified studies. From a very large pool of studies, 80 studies that met the defined inclusion and exclusion criteria were deemed most relevant to the aim of the review and included in the relevant sections.

The entire process of this narrative review involved identifying key information from each study, such as objectives, methodology, results, and conclusions. The findings were organized into thematic sections of the review and integrated into a coherent narrative highlighting the key areas where personalized psycho-oncology complements cancer care.

## 3. Relevant Sections

Based on the analysis of the current literature, the following sections describe the main phases in which psycho-oncology complements cancer treatment and the related disciplines that characterize these phases.

### 3.1. Psycho-Oncological Screening and Diagnosis

In the following paragraphs, we discuss the benefits of psychological screening and the assessment of mental health symptoms or conditions in oncology, along with the available tools suitable for these purposes.

In general, personalized screening for psychological distress has been shown to be crucial for understanding the psychological consequences of cancer, both at diagnosis and in the long-term follow-up of various types of neoplasms (e.g., breast, lung, and neurological cancers). Furthermore, early detection of stress coupled with tailored, personalized interventions can prevent the escalation of stress, enhance quality of life, and lower healthcare costs [16,17].

The literature shows a moderate impact on psychological well-being [18], but appropriate, individualized screening can lead to appropriate treatment [19]. Review of studies looking at the impact of personalized psychological care shows that systematic, tailored screening for distress leads to positive outcomes following psycho-oncological intervention [20]. An observational study highlighted that testing should be complemented by a psychological interview to detect false negatives, underscoring the need for individualized assessment. In addition, psychological needs may be important even when there is no overt distress [21]. A cross-sectional study conducted with survivors in ambulatory care suggests that brief psycho-oncological screening with self-reporting can be integrated into routine care to promote an individualized discussion between patient and physician about symptoms [22].

Interestingly, a randomized trial was proposed in 2014 that investigated a personalized stepwise model combining screening, medical consultation and professional psycho-oncological care in a structured manner. It included a three-stage personalized screening: analysis of distress, feedback of screening results to the physician, and consultation with the patient, and possible referral to a counseling center based on a shared decision between patient and physician [23]. The personalized stepped care model showed better results for referral to counseling. Although emotional distress was not significantly improved, the perception of the quality of care improved and the utilization of outpatient psychiatric help increased in patients with psychiatric/psychological comorbidity and decreased in those without. Furthermore, when multidisciplinary, tiered screening included the subsequent provision of personalized social services, financial hardship was more effectively managed [23,24,25].

Although there is little specific literature on personalized psychiatric/psychological screening in psycho-oncology, valuable insights can be found in international clinical guidelines [26,27]. Although the concept of “personalization” is not explicitly emphasized in these guidelines, the possibility of tailoring the screening process to individual needs emerges as a background concept. The process begins with the careful selection of healthcare professionals. According to the documents cited, nurses are typically appropriate for initial screening due to their frequent and direct interaction with newly diagnosed patients. As the patient enters the survivorship phase, the role shifts to the general practitioner, who is better suited for follow-up care with their extensive knowledge of the patient’s long-term health status.

The specialized training of these professionals is also crucial. As described in recent research [28], this training is tailored to enhance their ability to make sensitive and empathetic assessments and equip them with the necessary skills to manage the complex emotional responses of patients from different cultural backgrounds. This will ensure that screenings are conducted respectfully and effectively and truly reflect the individual’s mental state.

Numerous self-report screening questionnaires are available to efficiently identify each patient’s specific conditions, signaling the need for subsequent evaluation by trained clinicians, such as psychologists or psychiatrists. These questionnaires explore various aspects and constructs, offering mental health insights that range from assessing general distress or psychological symptoms to identifying cutoff-based psychopathological indicators that necessitate intervention. For those focusing on general distress, the Distress Thermometer (DT) is commonly used, whereas for identifying potential psychopathology that requires further evaluation, the Hospital Anxiety and Depression Scale (HADS) is the most prevalent tool. The HADS and the Distress Thermometer, both screening instruments for emotional distress, have been shown to be more effective than the Edmonton Symptom Assessment System-revised (ESAS-r) in assessing psychological and psychiatric symptoms [29]. These instruments are designed to easily, quickly, and sensitively detect levels of anxiety and depression, making them particularly useful in routine clinical practice.

Finally, self-report tools like the Patient Health Questionnaire-9 (PHQ-9) exhibit high sensitivity and specificity in identifying the likelihood of major depressive episode, utilizing DSM criteria-based algorithms.

To integrate these self-report tools effectively into routine clinical practice, a sequential approach can be adopted. Initially, the outpatient visits can begin with broader and simpler tools like the DT and HADS to assess general distress, anxiety, and depression. If these initial assessments reveal significant cutoffs, clinicians can then proceed to more specific tools, like the PHQ-9. This sequential use of questionnaires ensures a comprehensive evaluation of the patient’s mental health, allowing clinicians to detect any signs of psychopathology that may require further investigation.

If the PHQ-9 or other specific tools indicate the need for a more detailed evaluation, clinicians can then utilize specialized diagnostic tools such as the Mini International Neuropsychiatric Interview (MINI). The MINI provides a structured, reliable method for diagnosing psychiatric/psychological disorders based on DSM criteria. With the help of this interview, disorders such as major depression, anxiety disorders, and other psychiatric/psychological comorbidities can be identified.

From a broader perspective of the patient’s life, it is also beneficial to include questionnaires assessing quality of life. The EORTC QLQ-C30 (European Organization for Research and Treatment of Cancer Quality of Life Questionnaire) and its specific modules are comprehensive instruments for evaluating the quality of life in cancer patients. These questionnaires assess multiple dimensions of a patient’s experience, including physical, emotional, and social functioning, and are widely recognized for their sensitivity and specificity in representing health-related quality of life. Similarly, the World Health Organization-5 Well-Being Index (WHO-5) is a brief, self-reported measure of current psychological well-being that has been validated in various patient populations, including cancer patients.

For patients undergoing hematopoietic cell transplantation, pre-transplant psychological screening is recommended using instruments such as the Psychological Adjustment to Illness Scale (PAIS), the Beck Depression Inventory-Fast Screen (BDI-FS), the Brief Symptom Inventory-18 (BSI-18) and the Substance Abuse Subtle Screening Inventory (SASSI). These instruments help to assess psychological readiness and potential risk factors that could influence the outcome of transplantation.

In genetic counseling, research suggests that in addition to psychopathology, exploring other needs and existential concerns can greatly enhance psychological support during counseling. For example, Vos et al. [30] highlight that addressing issues such as fear of cancer recurrence, family dynamics, and personal meaning can increase the effectiveness of genetic counseling sessions for hereditary breast/ovarian cancer.

In summary, the use of these diverse and tailored screening tools underscores the importance of a personalized approach to psycho-oncology. By accurately assessing and addressing the individual psychological needs of each patient, healthcare providers can significantly improve the overall quality of care and outcomes for people with cancer.

In terms of diagnosis, it is important to highlight that, beyond the psychiatric conditions coded by DSM-5-TR and ICD-11, there are additional emotional and psychopathological aspects that do not perfectly align with these diagnoses but are equally important for the comprehensive care of cancer patients [31,32].

In addition, psychiatric/psychological disorders in cancer patients are often considered only as adjustment disorders due to the significant stress involved. While these disorders do occur, it is critical to accurately diagnose psychiatric/psychological disorders, as many symptoms may indicate major depressive disorder or another psychiatric disorder rather than adjustment disorder. Accurate diagnosis requires clinicians to be able to distinguish between adjustment disorders and other psychiatric/psychological syndromes through careful assessment [33].

To improve this process, the Diagnostic Criteria for Psychosomatic Research (DCPR) framework is crucial for translating psychosocial factors into operational diagnostic tools. It provides criteria for syndromes such as demoralization, abnormal illness behavior, and alexithymia and allows a deeper understanding of the way the patient perceives and responds to their illness. The use of DCPR helps identify these conditions, which are often overlooked by DSM-5-TR and ICD-11 and allows for a more individualized and effective diagnosis and intervention strategy [34].

A personalized diagnosis also requires distinguishing between symptoms that are due to a medical condition (e.g., pain, loss of appetite) and those that have a psychiatric/psychological origin. An accurate assessment helps to understand whether a symptom such as fatigue is primarily due to cancer or secondary to a depressive disorder [35]. In addition, biological components, especially inflammatory markers, should be considered in the diagnostic process. Different levels of inflammation can influence the onset of psychiatric/psychological symptoms and lead to subtypes of depression. Understanding these biomarkers helps to tailor psychiatric/psychological assessments and interventions [36].

Considering all these aspects, a founded approach focused on patient should encompass comprehensive and personalized psycho-oncological care that integrates psychological screening with tailored interventions. This includes utilizing validated screening tools such as the Distress Thermometer and HADS to identify distress at various stages of cancer treatment, with an emphasis on early detection and personalized interventions to reduce stress and improve quality of life. Screening should be complemented by psychological interviews to detect false negatives and ensure that non-overt distress is managed, fostering open communication between patients and physicians for individualized care. Implementing structured stepwise care models, providing specialized training for healthcare professionals, and adapting screening tools to individual patient needs are crucial [37].

### 3.2. Psycho-Oncology and Treatment

Some comprehensive reviews in the literature deal with psycho-oncological interventions. For example, the study by Turchi et al. provides a comprehensive overview of mental health interventions for cancer patients [38]. Our review aims to go a step beyond these existing studies by placing greater emphasis on the concept of personalization of treatment. We aim to demonstrate how tailored psychological and psychiatric care can better meet the specific needs of these patients.

#### 3.2.1. Psychological Treatment

##### Individual Therapy

- Cognitive Behavioral Therapy (CBT) and Its Adaptations

Cognitive behavioral therapy (CBT) is a basis for psychological interventions in oncology. CBT combines cognitive and behavioral techniques to help patients cope with distressing thoughts and behaviors. Studies have shown that CBT can effectively reduce anxiety, depression, and stress in cancer patients [39].

The strength of CBT lies in its adaptability and its ability to be tailored to the individual needs of cancer patients. Personalization in CBT means that therapy sessions are tailored to the specific cognitive and behavioral challenges of individual patients. For example, a patient suffering from severe fear of cancer recurrence might benefit from cognitive restructuring techniques to help them challenge and change their catastrophic thoughts about recurrence. This process involves identifying negative thought patterns and replacing them with more realistic and positive ones, thereby reducing anxiety and improving emotional well-being [40].

Another aspect of personalization in CBT is the inclusion of patient-specific goals and preferences in the treatment plan. During the initial assessment, therapists work with patients to identify their main concerns and treatment goals. This collaborative approach ensures that therapy is relevant and directly addresses the issues that are most important to the patient [41]. For example, some patients may be focused on coping with chemotherapy-induced fatigue, while others may be focused on coping with the emotional impact of a new diagnosis. By tailoring therapy to the patient’s goals, CBT becomes more effective and responsive [39,40,41].

In addition, CBT can be adapted to include other therapeutic elements that are consistent with the patient’s cultural and personal background. Thus, the integration of mindfulness practices into CBT may be particularly beneficial for patients who find support in meditative practices. This integration may help patients to develop a more holistic approach to coping with stress and anxiety by combining cognitive restructuring with mindfulness-based strategies to improve overall treatment effectiveness [42].

Personalization also extends to the format and delivery of CBT. Thanks to advances in technology, CBT can be delivered in a variety of ways, such as face-to-face sessions, teletherapy, and even via digital platforms. This flexibility allows patients to choose the format that best suits their lifestyle and preferences and ensures that they adhere better to the treatment plan. For example, a patient who has difficulty getting around or has a busy schedule might prefer teletherapy sessions, which can be just as effective as in-person therapy [43,44].

- Mindfulness-Based Interventions

Mindfulness-based interventions (MBIs) have gained popularity in psycho-oncology as they focus on present-moment awareness and non-judgmental acceptance of thoughts and emotions. Mindfulness-based stress reduction (MBSR) and mindfulness-based cognitive therapy (MBCT) are two widely used MBIs. These interventions have been shown to reduce symptoms of anxiety and depression, improve quality of life, and enhance emotion regulation in cancer patients [45].

In a study by Baydoun et al., MBSR was found to significantly reduce fatigue and emotional distress in cancer patients, highlighting its potential to treat both psychological and physical symptoms of cancer [46]. MBCT, which combines mindfulness practices with cognitive therapy, has also been shown to be effective in reducing fear of recurrence and improving psychological well-being [47].

Personalization of MBI involves tailoring mindfulness practices to the specific experiences and needs of cancer patients. For example, patients suffering from severe anxiety might benefit from mindfulness exercises that focus on grounding and breath awareness, while patients struggling with chronic pain might find body scan meditations more helpful. This individualized approach ensures that each patient receives the type of mindfulness practice that is most beneficial for their particular situation [48].

##### Personalized Stepped Care Psychological Interventions

Personalized stepped care psychological interventions are tailored to the individual psychological needs of cancer patients based on their specific emotional and psychological state. In this approach, patients are categorized into different levels of psychological distress and offered appropriate interventions [49].

Hao et al. [50] conducted a study on personalized stepped care psychological interventions in breast cancer patients, which showed a significant improvement in negative emotions and quality of life compared to routine care. Patients were divided into three grades based on their psychological assessment scores, and each group received targeted interventions. For example, patients with mild distress received basic supportive counseling, while patients with severe distress underwent intensive psychological therapy, including cognitive behavioral techniques and relaxation training.

The stepped care approach ensures that interventions are tailored to the severity of the patient’s psychological distress. This means that resources are used efficiently, and patients receive the care that is most likely to benefit them. For example, a patient with severe anxiety might receive more frequent and intensive therapy, while a patient with mild anxiety might benefit from less frequent, supportive counseling. This stratification ensures that patients do not receive a one-size-fits-all intervention, but a carefully tailored plan that meets their specific needs [51].

##### Group Therapies

Group therapy in psycho-oncology offers a useful approach to addressing the psychological needs of cancer patients. By providing a space in which individuals can share their experiences and support each other, group therapy can significantly improve emotional well-being and coping strategies [52].

The existing literature suggests that one of the main benefits of group therapy is that it fosters a sense of community and shared understanding among participants [53]. However, to maximize its effectiveness, it is crucial that group therapy sessions are tailored to the specific needs of the participants. Personalization in group therapy can be achieved in several ways:

1. Homogeneous group composition: most studies advocate the formation of groups based on specific characteristics such as cancer type, stage of treatment or demographic factors (e.g., age, gender) to ensure that participants share common experiences and challenges [54]. For example, research shows that women with breast cancer benefit more from a group that specifically addresses body image and femininity issues relevant to their experience [55].
2. Focus on common psychological problems: The literature shows that group therapy sessions can be designed to address specific psychological issues shared by participants. For example, groups that focus on anxiety, depression, or fear of relapse can provide targeted interventions that directly address participants’ needs. This approach not only increases the relevance of the therapy, but also increases the likelihood of positive outcomes [41].
3. Flexible therapy models: Introducing flexible therapy models that adapt the intensity and type of interventions to the evolving needs of the group is another effective personalization strategy. For example, some groups may benefit from more structured cognitive behavioral therapy (CBT) initially, followed by more open, supportive discussions as participants become more comfortable and better able to manage their symptoms.

Mindfulness-based intervention (MBI) groups focus on mindfulness in the present moment and non-judgmental acceptance of thoughts and emotions, which can help patients manage the psychological stress associated with cancer. Mindfulness-based arts interventions (MBAIs) combine mindfulness with art therapy, which includes music, theater, and expressive arts. These interventions have been shown to be very useful in reducing stress and negative emotional experiences while improving quality of life and overall well-being: studies by Rieger et al. [56] and Xunlin et al. [57], for example, have found that MBAIs help cancer patients to express their emotions more freely and connect with others facing similar challenges, leading to a reduction in psychological distress.

Music therapy, another non-pharmacological treatment, has shown significant effects in reducing depression and anxiety and positively influenced physiological parameters such as respiratory rate, blood pressure, and heart rate [58,59].

#### 3.2.2. Psychiatric Treatment

Psychiatric treatment in the context of oncology involves the management of psychiatric/psychological disorders that may arise as a consequence of cancer diagnosis and treatment. This section details the pharmacological interventions used to address conditions such as depression, anxiety, and adjustment disorders among cancer patients. It covers the use of antidepressants, anxiolytics, mood stabilizers, and antipsychotic medications, discussing their efficacy, potential side effects, and the considerations necessary for their use in patients undergoing cancer treatment.

Effective integration of both psychotherapeutic and psychiatric interventions is essential for comprehensive cancer care, ensuring that patients receive holistic support that addresses both their psychological and psychiatric needs.

##### Antidepressants

As part of the personalized approach to antidepressant therapy in psycho-oncology, the strategic selection of SSRIs and other antidepressant classes involves careful consideration of the specific subtypes of depression and their accompanying symptoms, as well as the patient’s overall health profile. This decision-making process is recommended in the literature, including by Grassi et al. [60], who emphasize the importance of tailoring the pharmacodynamics and pharmacokinetics of antidepressants to the individual patient’s needs. For example, SSRIs such as citalopram or paroxetine are often preferred for the treatment of depression associated with anxiety symptoms or panic disorder due to their potent anxiolytic effects, in order to effectively alleviate the increased anxiety associated with a cancer diagnosis. On the other hand, sertraline and escitalopram are used for their activating properties in anergic depression, where patients suffer from significant lethargy. These SSRIs can be administered in higher doses to maximize their energizing effects and improve mood and vitality, which is crucial for patients struggling with cancer-related fatigue [61,62].

In addition, bupropion is recognized in the literature [63] for its efficacy on depressive symptoms without the sedative effects that can exacerbate anergy, making it a valuable option to increase engagement in daily activities.

The role of mirtazapine and SNRIs such as venlafaxine and duloxetine also play a crucial role in personalized antidepressant strategies. Mirtazapine is particularly beneficial for patients suffering from insomnia and significant weight loss due to its sedative and appetite-stimulating effects. This makes it an ideal choice to combat the cachexia commonly seen in cancer patients, as it addresses both psychological and physical health challenges, as noted in an article by Kim, Sung-Wan, et al. [64]. Most studies agree that SNRIs are effective due to their dual effects on depression and pain management [65]. Venlafaxine and duloxetine are not only effective in the treatment of mood disorders, but also provide significant relief from neuropathic pain, a common and debilitating symptom in cancer patients. In addition, venlafaxine is known for its effectiveness in treating hot flashes, particularly in breast cancer patients undergoing hormone therapy [66].

Vortioxetine is characterized above all by its favorable tolerability profile, which makes it a suitable choice for older adults. It is also a compelling option for younger patients, particularly those who are concerned about the sexual side effects often associated with other antidepressants, as vortioxetine tends to have a lower incidence of such adverse effects [67].

##### Anxiolytics and Hypnotics

In oncology patients, benzodiazepines play a crucial role in coping with various aspects that occur during cancer treatment. These drugs are primarily used to relieve acute anxiety associated with cancer diagnosis, treatment, or invasive procedures, making them essential in high-stress situations. They also help with insomnia by promoting relaxation and enabling restful sleep—common problems caused by anxiety, pain, or side effects of treatment. Benzodiazepines are effective in controlling anticipatory nausea in chemotherapy patients, especially when combined with antiemetics and behavioral techniques. In addition, drugs such as midazolam are used during invasive or stressful medical procedures due to their sedative properties. They are also used to treat the symptoms of delirium and severe agitation, especially in advanced stages of the disease, thus contributing to palliative care. In the complex landscape of psycho-oncology, the use of benzodiazepines requires a highly individualized approach in which efficacy and safety must be skillfully weighed against each other. The choice of benzodiazepines such as lorazepam and alprazolam are preferable due to their pharmacokinetic properties and potential for drug–drug interactions. These benzodiazepines are preferred because of their shorter half-life and lack of active metabolites, reducing the risk of daytime cumulation and sedation [68]. Dell’Osso et al. [69] add to this perspective by emphasizing the broader therapeutic and toxicity considerations of benzodiazepines in cancer treatment. They emphasize the fact that while benzodiazepines are generally safe, they have a narrower therapeutic window in cancer patients. This is of critical importance in the context of their interactions with other central nervous system depressants such as opioids, where sedative effects may be enhanced, increasing risks such as respiratory depression and profound sedation. In addition, this work suggests that benzodiazepines may impair cognitive function and potentially exacerbate symptoms of delirium, particularly in vulnerable groups such as the elderly or cognitively impaired. The therapeutic strategy therefore goes beyond the mere selection of a benzodiazepine. It involves an integrated approach that takes into account the health profile of each patient, including liver function and other concomitant diseases that may affect drug metabolism. The existing literature advocates the prudent use of benzodiazepines that are primarily metabolized by conjugation rather than oxidation, thereby minimizing the risk of adverse drug interactions [70].

##### Antipsychotics

Antipsychotic medications are very useful in the personalized treatment of psychiatric symptoms in oncology to overcome the complex challenges encountered in cancer treatment. These medications are used not only for their primary psychiatric indications but also to treat a spectrum of symptoms exacerbated by the cancer or its treatment, including delirium, severe anxiety, and sleep disturbances. The literature highlights the efficacy of various antipsychotics in relieving agitation and confusion during delirium, particularly in palliative care. According to a recent study [71], haloperidol is particularly favored for rapid control of severe symptoms, although its use requires careful monitoring due to potential side effects such as extrapyramidal symptoms and QT prolongation. Olanzapine, which is known for its efficacy and less severe side effects than haloperidol, is also suitable for the treatment of chemotherapy-induced nausea and vomiting due to its antiemetic properties. Caution should be exercised when using this medication in patients with respiratory insufficiency as it can worsen lung function. For the same reason, it should not be used together with benzodiazepines and opioids with a long half-life, which are frequently used in this area. Quetiapine is particularly suitable for patients with sleep disorders and anxiety because of its sedative effect. It helps to stabilize mood and improve cognitive function, which is essential for patients struggling with a mixture of depressive and psychotic symptoms during cancer. Aripiprazole is characterized by its minimal sedative and metabolic effects, making it a valuable option for patients who are already struggling with weight and metabolic issues due to their cancer treatment. Its potential to act as a stimulant is beneficial in the treatment of hypoactive delirium, as reported in a comprehensive review by Thekdi et al. [72]. The choice of an antipsychotic in cancer treatment is also strongly influenced by the patient’s general state of health, existing comorbidities, and specific clinical symptoms. For example, while clozapine is effective in treatment-resistant psychiatric disorders, its use is contraindicated in patients with hematologic diathesis due to an increased risk of malignancies (lymphoma and leukemia), which is emphasized by Tiihonen et al. [73].

Careful consideration of the pharmacological profile of each antipsychotic against the background of a patient’s specific cancer type, treatment phase, and potential drug interactions is essential. This approach ensures that the therapeutic benefits of antipsychotics are maximized while minimizing adverse effects to improve patients’ overall success in cancer treatment. The most important interaction of this class of drugs is examined in more detail in a separate section.

##### Mood Stabilizers

Mood stabilizers in psycho-oncology can be carefully tailored to a wide range of emotional and neurological complications that may arise from cancer or its treatment, underscoring the importance of a personalized therapeutic strategy. For example, although lithium is traditionally used to treat bipolar disorder, its use in oncology is viewed with caution due to potential side effects. However, according to a recent study, lithium can be used in certain cases to reduce chemotherapy-induced neutropenia due to its granulocyte-stimulating properties [74] or as a neuroprotective agent against chemotherapy-induced cognitive dysfunction, as discussed by Khasraw et al. [75]. Valproic acid (VPA) and carbamazepine, typically used to control seizures, are also used in the treatment of mood disorders and neuropathic pain common in cancer patients [76]. Valproic acid is particularly known for its mood-stabilizing effects, which are helpful in managing impulsivity and disinhibition that may be exacerbated by steroids or the stress of a cancer diagnosis. Gabapentin and pregabalin are valuable because they can relieve neuropathic pain and hot flashes, especially in patients undergoing hormone therapy [77]. Lamotrigine, although less commonly used, offers potential benefits in neuropathic pain and fits into personalized treatment strategies that take into account the specific symptom profile and side effect tolerance of individual patients. Similarly, the benefits of topiramate extend beyond seizure prophylaxis to the treatment of migraine and neuropathic pain and offer a comprehensive approach to symptom control in cancer patients with certain comorbidities such as obesity or binge eating [78].

##### Drug–Drug Interactions

In the context of psycho-oncology, personalized treatment plans must carefully consider potential drug–drug interactions to optimize therapeutic outcomes and minimize adverse effects. Cancer patients often require complex drug regimens that include chemotherapeutic agents, psychotropic drugs, and supportive medications. The interaction between these drugs can significantly impact both efficacy and safety, emphasizing the need for careful monitoring and management. A comprehensive review by Yap and other authors [79] provides us with a very useful and practical understanding of the potential interactions between psychiatric and oncologic medications.

Alkylating agents, such as cyclophosphamide and ifosfamide, can inhibit the enzyme CYP2B6. This inhibition may reduce the clearance of bupropion, a major substrate of the same enzyme, potentially altering its clinical efficacy. Therefore, monitoring of patient response to bupropion is essential when these drugs are co-administered. In addition, both cyclophosphamide and ifosfamide are important substrates of CYP3A4. When combined with fluvoxamine, a CYP3A4 inhibitor, their plasma concentrations may increase, increasing the risk of toxicities such as bone marrow suppression. Caution should therefore be exercised with such combinations [80].

In addition, clozapine may interact with various alkylating agents such as cyclophosphamide, dacarbazine, and melphalan, leading to an increased risk of additive myelosuppression. To mitigate these risks, clozapine should not be used concomitantly with these agents as the potential for hematologic toxicities may be increased [81]. Naltrexone also carries an increased risk of hepatotoxicity when used concomitantly with carmustine and dacarbazine. In view of the serious liver damage, concomitant use of these drugs is generally not recommended unless absolutely necessary, and the patient’s liver function should be closely monitored [82].

Procarbazine, an alkylating agent with weak MAO inhibitor activity, may interact with several antidepressants, including tricyclic antidepressants (TCAs), selective serotonin reuptake inhibitors (SSRIs), and serotonin/norepinephrine reuptake inhibitors (SNRIs). These interactions may lead to central nervous system (CNS) toxicity or serotonin syndrome, which is characterized by mental status changes, myoclonus, hyperthermia, and autonomic instability. Due to these potential interactions, concomitant administration of procarbazine with these psychotropic drugs is not recommended, and a sufficient interval should be observed between discontinuation of these drugs and initiation of procarbazine therapy [83].

Among the antimetabolites, the clearance of pemetrexed, which is mainly excreted via the kidneys, can be reduced by nephrotoxic drugs such as lithium. This interaction can lead to increased plasma concentrations of pemetrexed, necessitating close monitoring of renal function [84]. Methotrexate, another antimetabolite, has been associated with an increased risk of haloperidol-induced photosensitivity [85]. Patients taking methotrexate should avoid sunlight or bright light to avoid photosensitive reactions.

Antimicrotubules, including the taxanes docetaxel and paclitaxel, are substrates of CYP3A4. Psychotropic drugs that inhibit this enzyme, such as fluoxetine, fluvoxamine, and bromocriptine, may alter plasma concentrations of taxanes, necessitating close monitoring of dose-related toxicities. Similarly, fluvoxamine may inhibit CYP3A4 activity and thus increase plasma concentrations of vinca alkaloids such as vinblastine and vincristine, which may lead to neurotoxicity. Therefore, patients taking these combinations should be monitored for signs of neurotoxicity [86].

Regarding biological modifiers, aldesleukin (IL-2) has been investigated for its potential interaction with naltrexone, which could increase the risk of hepatotoxicity. The concomitant use of IL-2 and naltrexone should be approached with caution, with regular monitoring of liver function [87]. Interferon alfa, another biological modifier, may increase the risk of peripheral neuropathy when combined with disulfiram. Monitoring for symptoms of neuropathy and adjusting the dose accordingly is crucial.

Corticosteroids, which are frequently used in cancer therapy, are metabolized by CYP3A4. Co-administration with CYP3A4 inhibitors or inducers may alter their plasma concentrations and requires careful monitoring. Dexamethasone and prednisone may also induce CYP450 isozymes, potentially reducing plasma concentrations of psychotropic drugs metabolized by these enzymes. In these cases, monitoring and possible dose adjustments are required [88].

Hormone antagonists such as tamoxifen rely on active metabolites formed via CYP2D6. SSRIs such as paroxetine can inhibit this enzyme and thus reduce the efficacy of tamoxifen. Careful consideration and monitoring are necessary when taking these drugs together, although recent reviews do not agree on a potential harmful effect of this combination [89].

Platinum drugs such as cisplatin and oxaliplatin have been reported to interact with lithium, which may alter lithium levels and increase the risk of nephrotoxicity. Careful monitoring of renal function and lithium levels is essential [90]. Clozapine should not be used concomitantly with platinum agents due to the risk of additive myelosuppressive effects [81].

Thalidomide, which is known for its CNS depressant effects, requires caution when co-administered with other CNS depressant agents such as hypnotics, anxiolytics, antipsychotics, antidepressants, and lithium. Monitoring for excessive CNS and respiratory depression is recommended.

Topoisomerase inhibitors such as irinotecan may interact with citalopram, reducing its clearance and increasing the risk of rhabdomyolysis [91]. Monitoring for signs of myopathy is essential.

Finally, tyrosine kinase inhibitors such as imatinib may interact with psychotropic drugs metabolized by CYP3A4/5, 2C9, and 2D6 and increase their plasma concentrations [92]. It is important to pay attention to side effects and adjust the dose if necessary. Erlotinib and gefitinib may decrease the therapeutic response to midazolam by inducing CYP3A4, while gefitinib may increase plasma concentrations of atomoxetine, requiring close monitoring for signs of toxicity [93].

Although there are numerous data on the co-use of cancer treatments and psychotropic drugs, there is a notable lack of information on the interactions between newer innovative cancer treatments such as checkpoint inhibitor immunotherapies, Antibody-Drug Conjugates (ADC) drugs, novel targeted agents, and psychotropic drugs. Theoretically, there should be no significant pharmacodynamic and/or pharmacokinetic interactions between these treatments. However, our current understanding of these interactions is limited, and it is imperative that we recognize that we need to address these combined treatment strategies soon.

### 3.3. Psycho-Oncological Follow-Up

Personalizing psycho-oncological follow-up involves understanding the needs of each individual patient and adapting the care offered to their evolving needs. This process involves several critical components, each of which is supported by the existing literature, as detailed in this paragraph. The stepped care approach, already used in other phases of illness, is fundamental to tailoring follow-up care based on the severity of psychological and psychiatric illness. Studies show that this approach can range from minimal support for patients with mild symptoms to intensive, specialist-led follow-up for patients with severe symptoms [18]. By using the same diagnostic tools used in the initial assessment, healthcare providers can closely monitor changes in the patient’s symptom profile over time. Regular monitoring of symptom progression allows for timely adjustment of interventions and ensures that patients receive the right level of care at each stage of their treatment. This dynamic “monitoring and adjustment” is essential for maintaining the effectiveness of the therapeutic approach and improving the overall outcome for the patient.

Another important aspect of personalized psychological/psychiatric aftercare is the incorporation of patient-reported outcome measures (PROMs) into routine assessments. PROMs provide real-time insights into psychological/psychiatric symptoms and treatment responses and allow patients to share their experiences and symptoms directly. This patient-centered method of data collection allows clinicians to tailor their treatment strategies more effectively. For example, PROMs can help identify specific areas of concern, such as depression, anxiety, or fatigue, which can then be addressed through targeted interventions. By focusing on these individualized needs, healthcare providers can significantly improve patients’ quality of life and overall well-being [94].

Flexible treatment plans are essential to address the dynamic nature of psychological and psychiatric symptoms in cancer patients. Regular follow-ups allow clinicians to adjust medication dosages or switch to alternative therapies if patient feedback, side effects, or potential drug interactions dictate. The literature emphasizes the importance of this flexibility to ensure that treatments remain effective and tolerable for patients [95]. By continuously assessing and adjusting treatment plans, clinicians can better respond to the changing needs of their patients and thus improve therapeutic outcomes.

Interdisciplinary coordination is critical for effective psychiatric/psychological follow-up in psycho-oncology. Seamless collaboration between oncologists, psychiatrists, psychologists, primary care physicians, and other specialists ensures continuity of care and comprehensive treatment. Studies emphasize that this holistic, team-based approach allows healthcare professionals to share insights and adapt interventions to the evolving needs of the patient [96]. This coordinated approach not only improves patient outcomes but also the overall quality of care as each specialist contributes their expertise to a unified treatment strategy.

Psycho-oncological follow-up aims to recognize distress during routine examinations and to interpret relative results accurately. It also aims to combat the fear of cancer recurrence, a common concern among survivors. By integrating personalized strategies and maintaining interdisciplinary care, psycho-oncology follow-up can significantly improve the psychological well-being of cancer patients. This comprehensive approach ensures that patients receive ongoing, tailored support throughout their cancer journey that addresses both their physical and psychological needs.

### 3.4. The Importance of the Inflammation in Personalized Psycho-Oncology

The pathophysiological mechanisms underlying the psychological distress of cancer patients are complicated and not yet fully understood. Genetic predispositions play a crucial role, as certain genetic variations may increase susceptibility to the development of psychological or psychiatric problems and influence an individual’s response to stress [97]. Metabolic changes also have a significant impact on mental health, as they disrupt hormonal and energy balance. Cancer and its treatments often lead to metabolic changes that affect mood and cognitive function, contributing to feelings of distress and anxiety [98].

In addition, the immunological condition can exacerbate psychological distress by affecting the central nervous system and neurochemical pathways. Among these factors, chronic inflammation stands out as a critical factor that is closely linked to both cancer progression and psychological distress, and its extent can vary considerably depending on the type of neoplasm. Lung cancer and prostate cancer, for example, can have different inflammatory profiles, which in turn can affect patients’ psychological/psychiatric symptoms [99]. Chronic inflammation, often characterized by elevated levels of C-reactive protein (CRP), has been associated with increased symptoms of depression and anxiety, suggesting that the management of inflammation may be an important component of psycho-oncology care [100].

The existing literature suggests that anti-inflammatory treatments, lifestyle changes to reduce stress, and dietary adjustments could potentially alleviate psychological symptoms by addressing the underlying inflammation. Personalization of pharmacological treatment requires consideration of inflammation levels, as the existing literature clearly demonstrates the association between inflammation and depressive symptoms in cancer patients [101]. For example, McFarland et al. highlight that elevated levels of inflammatory markers such as interleukin (IL)-6, tumor necrosis factor (TNF), and CRP are consistently associated with depressive symptoms in various types of cancer [36]. This suggests that depression in cancer patients can be categorized based on inflammatory profiles. Conventional antidepressants that target serotonin, such as selective serotonin reuptake inhibitors (SSRIs), may be less effective in patients with high levels of inflammation, whereas drugs that affect dopamine pathways may have better outcomes in patients with elevated inflammatory markers. In addition, the use of anti-cytokine agents, including TNF antagonists such as infliximab, has been associated with a reduction in depressive symptoms in treatment-resistant depression, provided that inflammatory biomarkers are elevated at baseline. Integrating psychological assessment with inflammation analysis helps to create comprehensive treatment plans that address both mental and physical health. By routinely testing inflammatory markers, patients with elevated IL-6, TNF, or CRP levels can be identified, allowing physicians to customize their treatments more effectively. While this approach offers promising personalized treatment strategies, there are some challenges to overcome. The process requires significant resources, including specialized laboratory equipment and trained personnel. In addition, the costs associated with frequent testing may not be covered by all healthcare systems. Integrating biomarker testing into existing clinical workflows is complex and requires changes in practice and increased coordination between healthcare providers. Despite these challenges, integrating personalized approaches to psycho-oncology that emphasize the importance of biomarkers and inflammation management can significantly improve patient outcomes.

Regular screening, personalized treatment plans, and continuous monitoring are essential components of this approach. The limitations of current diagnostic criteria will be further refined through ongoing research so that patients ultimately benefit from more targeted and effective interventions tailored to the specific cancer type and associated inflammatory and psychological/psychiatric profiles.

Therefore, considering the type of neoplasm, its histology and molecular mechanisms, also considering molecular impact of chemo/immuno-therapy or inflammation consequences after a surgical treatment, can lead health professionals to a proper psycho-oncological evaluation.

## 4. Discussion

With this narrative review, we aimed to provide a comprehensive overview of the current state of the personalized approach in psycho-oncology and to synthesize the diverse literature, providing a general background that we hope will be useful for future research.

However, the general limitations of the narrative approach must be kept in mind [102]. A narrative review does not provide an exhaustive, evidence-based synthesis for focused questions, may not include all relevant literature on a topic, and does not provide definitive guideline statements. Furthermore, while the authors have provided considered interpretations, the selection and analysis of the literature has been influenced by their own perspectives, meaning that different authors may derive different findings from the same body of research. Therefore, future systematic reviews are warranted to provide insights on specific and narrowly defined topics in this broad field of psycho-oncology.

With these limitations in mind, we found that although the field of psycho-oncology has made significant progress in integrating personalized approaches into the treatment of cancer patients, much remains to be done.

This review presents several personalized interventions, including psychotherapies and psychiatric treatments, all of which have significant potential to improve the psychological well-being of cancer patients. These interventions are not only effective but also adaptable, allowing for a high degree of personalization based on individual patient needs and preferences. The stepped care approach is particularly noteworthy as it provides an appropriate level of care depending on the severity of psychological symptoms. Research shows that this model not only improves mental health outcomes but also optimizes the use of health resources by ensuring that patients receive the right level of intervention at the right time [23,24]. The use of patient-reported outcome measures (PROMs) further enhances this approach by providing real-time insights into patient experience, enabling timely adjustment of care strategies [94]. The integration of inflammatory biomarkers into psycho-oncology represents a promising but difficult challenge. Studies have consistently linked chronic inflammation to depressive and anxiety symptoms in cancer patients [36]. By tailoring treatments to manage inflammation, it may be possible to alleviate some of the psychological distress associated with cancer. Interdisciplinary collaboration remains a cornerstone of personalized psycho-oncology. Effective communication and coordination between oncologists, psychiatrists, psychologists, and general practitioners is essential for comprehensive treatment. This collaborative approach ensures that all aspects of a patient’s health are considered, leading to better outcomes and higher quality of care [96].

## 5. Conclusions

Despite this progress, there are still some challenges in the implementation of personalized psycho-oncology. The integration of inflammatory biomarkers into personalized treatment plans is a promising area but requires significant resources and specialized training. The costs associated with frequent biomarker testing and the complexity of integrating such tests into clinical workflows present significant hurdles. In addition, current diagnostic criteria such as DSM-5-TR and ICD-11 often fail to capture the full spectrum of psychiatric/psychological phenomena in cancer patients, indicating a need for customized diagnostic tools and approaches. In addition, the effectiveness of personalized psycho-oncological interventions may be limited by logistical challenges such as the availability of trained professionals and the need for coordinated care across multiple disciplines.

One of the biggest challenges for the future is tailoring treatments to the specific type of neoplasm. Different types of cancer can lead to different symptoms depending on the organ affected, requiring highly specialized and individualized treatment approaches. For example, the psychological/psychiatric effects of breast cancer may differ significantly from those of lung or prostate cancer, requiring different therapeutic strategies. The development of clear guidelines differentiated by different neoplasms and comorbidities such as anxiety, depression, stress, and post-traumatic stress disorder will be crucial to advance personalized psycho-oncology.

Future research should focus on refining personalized strategies, developing accurate diagnostic tools, and evaluating the cost-effectiveness of various interventions. By addressing these challenges, psycho-oncology can evolve and provide more targeted and effective treatment to cancer patients. Ultimately, the aim is to improve not only the psychological well-being of patients but also their overall treatment outcomes, paving the way for a more comprehensive and personalized approach to cancer care.

## Data Availability

The original contributions presented in the study are included in the article; further inquiries can be directed to the corresponding author.
